# Determinants of waterpipe smoking in Iranian adults: results from the IROPICAN study

**DOI:** 10.3389/fpsyt.2023.1292503

**Published:** 2023-12-07

**Authors:** Monireh Sadat Seyyedsalehi, Aneri Shah, Hamideh Rashidian, Maryam Hadji, Maryam Marzban, Alireza Ansari-Moghaddam, Azim Nejatizadeh, Paolo Boffetta, Kazem Zendehdel

**Affiliations:** ^1^Department of Medical and Surgical Sciences, University of Bologna, Bologna, Italy; ^2^Cancer Research Center, Cancer Institute, Tehran University of Medical Sciences, Tehran, Iran; ^3^Icahn School of Medicine at Mount Sinai, New York, NY, United States; ^4^Health Sciences Unit, Faculty of Social Sciences, Tampere University, Tampere, Finland; ^5^Statistical Genetics Lab, QIMR Berghofer Medical Research Institute, Brisbane, QLD, Australia; ^6^Clinical Research Development Center, The Persian Gulf Martyrs, Bushehr University of Medical Science, Bushehr, Iran; ^7^Health Promotion Research Center, Zahedan University of Medical Sciences, Zahedan, Iran; ^8^Tobacco and Health Research Center, Hormozgan University of Medical Sciences, Bandar Abbas, Iran; ^9^Stony Brook Cancer Center, Stony Brook University, Stony Brook, NY, United States; ^10^Department of Family, Population and Preventive Medicine, Renaissance School of Medicine, Stony Brook University, Stony Brook, NY, United States; ^11^Cancer Biology Research Center, Cancer Institute, Tehran University of Medical Sciences, Tehran, Iran

**Keywords:** prevention, diseases, public health, tobacco, water pipe

## Abstract

**Introduction:**

Waterpipe smoking has become increasingly popular in Western countries, particularly among young individuals. This study aims to identify the factors influencing waterpipe smoking by focusing on consumption patterns.

**Methods:**

We utilized data from a multicenter case–control study (IROPICAN) conducted in Iran. Multivariate logistic regression estimated the adjusted odds ratio and 95% confidence intervals as a measure of association between waterpipe smoking and different factors.

**Results:**

Among 3,477 subjects were included, 11.8% were waterpipe smokers. Most of <50 years old smokers were occasional (80%), while daily smokers were often >50 years (85%). Around 59% of occasional users started it before 30 years old. Low education, low SES, alcohol consumption, cigarette smoking, secondhand smoke exposure, and opium use were associated with waterpipe smoking. Stratified analysis by frequency pattern showed an association between occasional smoking with age 0.97 (0.96–0.98), university degree 0.36 (0.17–0.76), urban dwellers 1.40 (1.06–1.86) and between high SES and daily smoking 0.34 (0.17–0.69).

**Conclusion:**

Our results offer valuable information to policymakers for developing waterpipe smoking control measures. The occasional waterpipe smoking results may be generalized to the younger people in Western countries.

## 1 Introduction

According to the World Health Organization (WHO), in 2023 tobacco smoking will be responsible for approximately 8.3 million deaths worldwide, with a significant impact observed in less-developed countries (1, 2). Waterpipe smoking, a traditional method of tobacco smoking originating from Southwest Asia and North Africa, has been increasing globally at a remarkable pace (3), particularly among young individuals (4–6). The highest prevalence of waterpipe smoking has been reported from countries located in the Eastern Mediterranean region, ranging from 2.5% in Oman to 37.2% in Lebanon (7). In Iran, waterpipe smoke is a significant public health concern, with prevalence reaching up to 10.9% in men and 16.8% in women, particularly in south and southeast Iran (8). Contrary to a slight decline in cigarette smoking over the past few years, waterpipe smoking has increased and is a serious problem, especially among young Iranian women and adolescents (9–12).

In this tobacco smoking method, known by many names such as hookah, shisha, narghile, hubble-bubble, and Kalyan in different cultures and countries, tobacco smoke passes through water, often infused with sweet and fruit flavors (1). Contrary to popular misconception that waterpipe smoking is less harmful than cigarette smoking, it contains several toxic chemicals and poses several health risks (13). The mixture contains more than 40 carcinogenic substances and metals like arsenic, cobalt, chromium, and lead (4, 14). Waterpipe smoking may lead to many health consequences such as lung and oral cancer, respiratory and coronary disorders (14, 15), low birth weight, sexual hormone disorders, and reproductive problems, particularly in women (1).

Numerous studies have examined and reported while personal, sociodemographic, and behavioral characteristics have been identified as factors influencing waterpipe smoking, it is important to recognize that these results are not uniform across various decades, cultural contexts, and geographical regions. There is a wide variation in the prevalence and patterns of waterpipe smoking across different geographical regions. Waterpipe smoking rates may increase in some countries and regions while declining in others. It is likely that this variation is caused by cultural differences, changing social norms, and the availability and accessibility of waterpipe smoking establishments. Understanding this variability is crucial when developing interventions, policies, and prevention strategies to effectively address waterpipe smoking (16–18). In the latest study reported in 2023 from Iran, prevalence of waterpipe smoking was associated with socioeconomic inequalities and lifestyle factors among adults. This study, however, did not provide any information regarding waterpipe metrics, particularly in terms of frequency patterns between daily and occasional users (19).

We aimed at identifying the factors associated with waterpipe smoking among Iranian men and women, and at exploring key waterpipe metrics in daily and occasional waterpipe smokers.

## 2 Methods

### 2.1 Study population and data collection

We analyzed data from the control group of IROPICAN project, a large multicenter case–control study conducted in Iran (20). The IROPICAN study was conducted between May 2017 and July 2020 to investigate the association between opium consumption and lung, colorectum, bladder, and head and neck cancer risk. The data was collected from general hospitals located in 10 provinces of Iran (Tehran, Fars, Mazandaran, Kerman, Golestan, Kermanshah, Khorasan-Razavi, Bushehr, Sistan and Baluchistan, and Hormozgan). A total of 3,233 healthy hospital visitors who were accompanying or visiting patients in non-oncology ward were selected as controls. The response rate in control group of the IROPICAN study was 89%.

### 2.2 Data collection

Trained interviewers employed standard data collection protocols to gather comprehensive information on detailed consumption of opium and tobacco products including cigarette, pipe, nass, and waterpipe. And also, other lifestyle factors such as education, asset ownership including vacuum cleaners, cloth washers, dishwashers, freezers, internet access, microwaves, laptops, mobile phones, cars, and ownership, job history. Data on starting age, stopping age, frequency and amount of use of each tobacco product was collected. Waterpipe smokers were asked about non-flavored (“tumbâk”) and flavored (“maassel”) tobacco. We defined frequency of smoking according to the daily or occasional (weekly or monthly) smoking and calculated amount of use by measuring the number of waterpipe heads smoked in each session. We divided the total number of waterpipe smoked by duration of smoking to derive the average amount of waterpipe smoked (heads per day). Furthermore, we defined a metric called head-year as the cumulative amount of waterpipe tobacco smoking by multiplying the total duration of waterpipe smoke by the average daily amount. A head year indicates smoking one waterpipe head per day for one year.

A principal component analysis was used to define the socioeconomic status (SES) of the participants, based on the number of years of education the participants had and asset ownership (21). Job history classified according to the seven different groups (Table 1).

**Table 1:** Waterpipe smoking status and selected demographic and lifestyle characteristics.

Characteristics	Non-waterpipe smokers *N* (%‡)	Waterpipe smokers		
		Ever smokers *N* (%‡)	Daily smokers *N* (%†)	Occasional smokers *N* (%†)
Total	3,068 (88.2)	409 (11.8)	149 (36.4)	260 (63.6)
Cigarette smoking, *N* (%)				
Never smoker	2,256 (90.24)	244 (9.76)	98 (40.16)	146 (59.84)
Regular smoker	812 (83.1)	165 (16.9)	51 (30.9)	114 (69.1)
Opium use, *N* (%)				
Never user	2,559 (88.8)	322 (11.2)	125 (38.8)	197 (61.2)
Non-regular user	119 (86.2)	19 (13.8)	3 (15.8)	16 (84.2)
Regular user	390 (85.1)	68 (14.8)	21 (30.9)	47 (69.1)
Alcohol use, *N* (%)				
No	2,956 (88.7)	377 (11.3)	137 (36.3)	240 (63.7)
Yes	112 (77.8)	32 (22.2)	12 (37.5)	20 (62.5)
Secondhand exposure to cigarette smoke, *N* (%)				
No	2,209 (90.3)	236 (9.6)	88 (37.3)	148 (62.7)
Yes	859 (83.2)	173 (16.8)	61 (35.3)	112 (64.7)
Age, *N* (%)				
<40	211 (82.1)	46 (17.9)	2 (22.4)	44 (77.6)
40–49	491 (87.8)	68 (12.2)	20 (29.4)	48 (70.6)
50–59	957 (89.4)	113 (10.6)	35 (31)	78 (69)
60–69	969 (88.7)	123 (11.3)	59 (48)	64 (52)
≥70	440 (88.2)	59 (11.9)	33 (55.9)	26 (44.1)
Gender, *N* (%)				
Female	957 (88.9)	120 (11.1)	48 (40)	72 (60)
Male	2,111 (88)	289 (12.0)	101 (34.9)	188 (65.1)
Province, *N* (%)				
Low prevalence regions	2,113 (93)	159 (7)	21 (13.21)	138 (86.79)
Tehran	731 (89.6)	85 (10.4)	9 (10.6)	76 (89.4)
Khorasan-Razavi	133 (78.2)	37 (21.8)	5 (13.5)	32 (86.5)
Kerman	504 (96)	31 (4)	5 (23.8)	16 (76.2)
Golestan	368 (98.4)	6 (1.6)	1 (16.7)	5 (83.3)
Mazandaran	132 (97.1)	4 (2.9)	1 (25)	3 (75)
Kermanshah	245 (97.6)	6 (2.4)	0	6 (100)
High prevalence regions	955 (79.25)	250 (20.75)	128 (51.20)	122 (48.8)
Fars	756 (80.2)	187 (19.8)	92 (49.2)	95 (50.8)
Bushehr	49 (58.3)	35 (41.7)	20 (57.1)	15 (42.9)
Hormozgan	66 (84.6)	12 (15.4)	6 (50)	6 (50)
Systan-Balouchestan	84 (84.0)	16 (16)	10 (62.5)	6 (37.5)
Rural residence, *N* (%)				
rural	1934 (89.2)	233 (10.7)	101 (43.3)	132 (56.6)
urban	1,134 (86.6)	176 (13.4)	48 (27.3)	128 (72.7)
Marital status, *N* (%)				
Married	2,782 (88.4)	365 (11.6)	137 (37.5)	228 (62.5)
Widow	162 (87.1)	24 (12.9)	10 (41.7)	14 (58.3)
Divorced/Separated	36 (87.8)	5 (12.2)	0	5 (100)
Single	78 (83.9)	15 (16.1)	2 (13.3)	13 (86.7)
Unknown	10 (100.0)	0	0	0
Education status, *N* (%)				
Illiterate	517 (87.6)	73 (12.4)	43 (58.9)	30 (41.1)
Diploma & less	2031 (87.3)	294 (12.6)	94 (32)	200 (68)
University	520 (92.5)	42 (7.5)	12 (28.6)	30 (71.4)
Socioeconomic status, *N* (%)				
Low	856 (87.9)	118 (12.1)	68 (57.6)	50 (42.4)
Medium	1,019 (86.7)	156 (13.3)	54 (34.6)	102 (65.4)
High	1,193 (89.8)	135 (10.2)	27 (20)	108 (80)
Employment status*, *N* (%)				
Group 1	360 (91.6)	33 (8.4)	9 (27.3)	24 (72.7)
Group 2	545 (88.2)	73 (11.8)	23 (31.5)	50 (68.5)
Group 3	517 (89.9)	58 (10.1)	26 (44.8)	32 (55.2)
Group 4	750 (85.8)	124 (14.2)	41 (33.1)	83 (66.9)
Group 5	84 (91.3)	8 (8.7)	2 (25)	6 (75)
Group 6	812 (87.8)	113 (12.2)	48 (42.5)	65 (57.5)

*Group 1 (professional, technical and related worker, administrative and managerial worker), group 2: (clerical and related workers, sales workers, service workers), group 3: (agricultural, animal husbandry and forestry workers, fishermen and hunters), group 4: (agricultural, animal husbandry, and farmer), group5: (production and related workers, transport), group 6: (military), and group 7: (other, including housewives, pension, unemployed). ^‡^Percentage over total number of subjects. ^†^Percentage over total number of waterpipe smokers.

### 2.3 Statistical analyses

According to the characteristics of the participants, frequencies were calculated for categorical parameters and mean, and standard deviations (±SD) were for continuous variables Waterpipe metrics were also reported by daily and occasional waterpipe smoking. We used unconditional logistic regression to estimate the adjusted odds ratio (OR) and 95% confidence intervals (CI) between never and ever waterpipe smoking and potential determinants overall and in daily and occasional smokers exclusively. All models were adjusted for age (continuous), gender, province, and SES (low, moderate, high level). All statistical analyses were carried out using Stata 14 (Stata Statistical Software: Release 14. College Station, TX: Stata Corp LLC).

## 3 Results

A total of 3,477 (2,400 men and 1,077 women) participants were included in our analysis, from which 409 were waterpipe smokers (11.8%) and 3,068 (88.3%) were non water pipe smokers. Numbers of ever-smokers for men and women were 289 (12.0%), and 120 (11.1%), respectively. Participants from Bushehr province had the highest proportion of waterpipe smokers (41.7%), followed by Khorasan Razavi (21.8) and Fars (19.8) provinces. The prevalence of ever waterpipe smoking was higher in individuals younger than 40 (17.9%), and those who were classified in the medium SES level (13.3%). Most of younger than 50 years old smokers were occasional smokers (80%), while daily smokers were often older than 50 years (85%). The prevalence of waterpipe smoking was higher among cigarette smokers 165 (16.9), opium users 87 (28.8), and secondhand cigarette smokers 173 (16.8), however there was no difference in occasional and daily waterpipe smokers. An additional analysis showed flavored tobacco smoking had a reverse association with age [<50 years = 59.46%, and ≥ 50 years = 40.53%] in our study.

Daily smokers used an average of more than 2 heads per day and smoked for a longer period than occasional users. Around 38% of water pipe users try it before 30 years old particularly among occasional users (59.6%) (Table 2). Consequently, they had a higher cumulative waterpipe smoking. It’s noteworthy that most non-favored (traditional) types of tobacco used by daily smokers 138 (92.6) whereas flavored tobacco which used by occasional users 77 (29.6).

**Table 2:** Waterpipe smoking metrics.

Water pipe metric	Waterpipe smoking		
	Ever, *N* (%)	Daily, *N* (%)	Occasional, *N* (%)
Total	409 (100.0)	149 (100.0)	260 (100.0)
Daily dose (head-day)			
<2	231 (56.5)	54 (36.2)	177 (68.1)
≥2	95 (23.2)	90 (60.4)	5 (1.9)
Unknown	83 (20.3)	5 (3.4)	78 (30)
Duration (years)			
<6 years	82 (20.05)	37 (24.8)	45 (17.3)
≥6 years	243 (59.4)	112 (75.2)	131 (50.4)
Unknown	84 (20.5)	0	84 (32.3)
Cumulative amount (head-years)			
< 5	143 (34.9)	16 (10.7)	127 (48.8)
5–20	86 (21.03)	42 (28.2)	44 (16.9)
>20	100 (24.4)	85 (57.05)	15 (5.8)
Unknown	80 (19.5)	6 (4.03)	74 (28.5)
Starting age (years)			
≥40	89 (21.8)	38 (25.5)	51 (19.6)
30–39	87 (21.8)	33 (25.5)	54 (20.8)
(20-)29	110 (26.9)	50 (33.6)	60 (23.1)
<20	45 (11)	28 (18.8)	17 (6.5)
Unknown	78 (19.1)	0	78 (30)
Cessation years			
Current	147 (35.9)	61 (40.9)	86 (33.1)
<10	91 (22.2)	39 (26.2)	52 (20)
≥10	97 (23.7)	49 (32.9)	48 (18.5)
Unknown	74 (18.1)	0	74 (28.5)
Tobacco type			
Flavored	88 (21.5)	11 (7.9)	77 (29.6)
Non-flavored	271 (66.3)	138 (92.6)	133 (51.1)
Unknown	50 (12.2)	0	50 (19.2)

Results of logistic regression showed inverse associations between ever waterpipe smoking and high SES (OR = 0.64, 95%CI 0.47–0.87), and university degree education (OR = 0.50, 95%CI 0.29–0.88) (Table 3). Waterpipe smoking was associated with alcohol drinking (OR = 2.61, 95%CI 1.68–4.08), ever cigarette smoking (OR = 2.47, 95%CI 1.91–3.18), secondhand smoking (SHS) (OR = 1.68, 95%CI 1.34–2.11), and regular opium use (OR = 2.35, 95%CI 1.70–3.25). There were no associations between waterpipe smoking and gender, marital status, and employment status.

**Table 3:** Characteristics associated with waterpipe smoking – results of multivariate logistic regression analysis.

Determinants	Ever waterpipe smoking	Daily waterpipe smoking	Occasional waterpipe smoking
	OR (95% CI)	OR (95% CI)	OR (95% CI)
Age			
<40	Ref.	Ref.	Ref.
40–49	0.49 (0.32–0.76)	2.09 (0.47–9.35)	0.43 (0.27–0.69)
50–59	0.43 (0.28–0.64)	1.80 (0.41–7.80)	0.39 (0.25–0.60)
60–69	0.41 (0.27–0.63)	2.34 (0.54–10.06)	0.32 (0.20–0.50)
≥70	0.36 (0.22–0.59)	2.03 (0.45–9.07)	0.27 (0.15–0.48)
(10-)yr increase in age	0.83 (0.74–0.92)	1.06 (0.88–1.27)	0.75 (0.66–0.84)
Gender			
Women	Ref.	Ref.	Ref.
Male	1.08 (0.84–1.38)	0.84 (0.57–1.26)	1.22 (0.90–1.66)
Location			
Low prevalence regions	Ref.	Ref.	Ref.
High prevalence regions	1.57 (0.86–2.86)	6.20 (2.39–16.1)	0.79 (0.32–1.91)
Education status			
Illiterate	Ref.	Ref.	Ref.
Diploma and less	0.90 (0.60–1.34)	0.79 (0.45–1.38)	0.80 (0.43–1.45)
University degree	0.50 (0.29–0.88)	0.71 (0.27–1.86)	0.36 (0.17–0.76)
Rural residence			
Rural	Ref.	Ref.	Ref.
Urban	1.22 (0.97–1.55)	0.99 (0.66–1.48)	1.40 (1.06–1.86)
Employment status*			
Group 1	Ref.	Ref.	Ref.
Group 2	1.17 (0.73–1.86)	1.08 (0.46–2.54)	1.17 (0.68–1.99)
Group 3	1.17 (0.70–1.94)	1.05 (0.43–2.55)	1.22 (0.66–2.23)
Group 4	1.53 (0.97–2.42)	1.38 (0.59–3.22)	1.56 (0.92–2.64)
Group 5	0.88 (0.38–2.03)	0.65 (0.13–3.21)	1.00 (0.38–2.61)
Group 6	1.61 (0.91–2.86)	2.03 (0.69–5.98)	1.39 (0.72–2.69)
Marital status^#^			
Married	Ref.	Ref.	Ref.
Single	1.13 (0.67–1.91)	0.62 (0.14–2.63)	1.25 (0.71–2.18)
Socioeconomic status			
Low	Ref.	Ref.	Ref.
Moderate	0.93 (0.70–1.24)	0.74 (0.44–1.24)	1.31 (0.90–1.91)
High	0.64 (0.47–0.87)	0.34 (0.17–0.69)	1.04 (0.70–1.56)
Alcohol			
No	Ref.	Ref.	Ref.
Yes	2.61 (1.68–4.08)	3.20 (1.59–6.45)	2.15 (1.27–3.64)
Cigarette smoking			
Never smoker	Ref.	Ref.	Ref.
Ever smoker	2.47 (1.91–3.18)	1.90 (1.26–2.87)	2.75 (2.03–3.73)
Opium use			
Never user	Ref.	Ref.	Ref.
non regular user	1.86 (1.08–3.22)	0.95 (0.28–3.23)	2.36 (1.30–4.27)
Regular user	2.35 (1.70–3.25)	2.31 (1.34–3.97)	2.42 (1.62–3.54)
Secondhand exposure to cigarette smoke			
No	Ref.	Ref.	Ref.
Yes	1.68 (1.34–2.11)	1.67 (1.16–2.42)	1.72 (1.31–2.26)

Adjusted by age, gender, province, and SES and each variable together if there wasn’t any correlation. *Group 1 (professional, technical and related worker, administrative and managerial worker), group 2: (clerical and related workers, sales workers, service workers), group 3: (agricultural, animal husbandry and forestry workers, fishermen and hunters), group 4: (agricultural, animal husbandry, and farmer), group5: (production and related workers, transport), group 6: (military), and group 7: (other, including housewives, pension, unemployed). ^#^Married, married and widow/single, single and divorced.

Stratified analysis by frequency pattern showed an inverse association between 10-yr increase in age and occasional smoking (OR = 0.75, 95%CI 0.66–0.84), high SES with daily smoking (OR = 0.34, 95%CI 0.17–0.69), and university degree with occasional smoking (OR = 0.36, 95%CI 0.17–0.76). There was a significant increase in occasional waterpipe smoking among urban dwellers (OR = 1.40, 95%CI 1.06–1.86). As expected, we identified an association between daily water pipe smoking and regions with a high prevalence of water pipe use (Fars, Bushehr, Systan-Balouchestan, and Hormozgan) (OR = 0.6.20, 95%CI 2.39–16.1) but not with occasional use. In both daily and occasional users, alcohol drinking, cigarette smoking, opium use, and secondhand smoking were similarly associated with waterpipe smoking (Table 3).

## 4 Discussion

This study was conducted to identify the factors associated with waterpipe smoking in a large sample of the Iranian population. Our results showed a direct association between waterpipe smoking and alcohol drinking, opium use, and cigarette smoking. Based on the frequency of smoking, daily smokers are mainly among people residing in high-prevalence areas who have been smoking waterpipe for an extended period. On the other hand, occasional smokers are more frequent people living in large cities and low-prevalence provinces. There may be a number of factors contributing to this; metropolitan residents tend to be younger, so they engage in tobacco use more for fun and are exposed to high-risk behaviors and lifestyles such as drinking alcohol and smoking cigarettes (22). The occasional smokers were younger and had lower education levels. Factors associated with their habit are probably more relevant to waterpipe smokers in low-prevalence countries, such as Europe and North America.

Previous studies reported that unhealthy lifestyles and behaviors are associated with waterpipe smoking (23). For instance, a survey from Lebanon revealed that waterpipe smoking was more prevalent among college students who binge drank alcohol more often (24). It has been found in previous studies that those with a positive attitude about cigarette smoking, drug use, or alcohol drinking are more likely to be curious about testing waterpipe, while those with a negative attitude are less likely to take it (25). On the other hand, some studies also suggest that the use of water pipes, which entails exposure to nicotine and other addictive chemicals may represent an encouraging factor for using another tobacco product or opioid in the future. Furthermore, it may influence dose or duration of use. There is, however, a need for further investigation and evidence to support this attitude (26).

According to our findings, there is a protective effect in association with higher education levels with occasional waterpipe smoking and among daily users with higher SES levels persons. Abdollahifard et al. reported that low education and low socioeconomic status were associated with waterpipe smoking in Shiraz, Iran (27), where daily waterpipe smoking is prevalence in men and women. Moghadam et al. in 2023 reported similar findings based on analyses of data one center of Persian cohort (19).

We found that most daily smokers prefer nonflavored or traditional tobacco, while occasional smokers, who are younger, prefer flavored tobacco. Previous studies showed that the sweet smell and taste of tobacco have an important role in increasing waterpipe smoking in the youth in many countries, and it is one of the reasons for starting and continuing waterpipe smoking, particularly on social events (27–30). Also, we found most occasional users try the water pipe use for the first time at the age of less than 30. Compared to daily smokers, occasional smokers used lower doses for a longer period of time.

We did not find an association between waterpipe smoking and employment status and marital status. Previous studies have shown inconsistent results regarding these two factors (23, 31). This heterogeneity can be related to several factors, such as the distribution of age and gender of the study population, family and social responsibilities, culture, and geographical location (23, 27, 32). Patterns of waterpipe smoking were not different between men and women: this can be explained by the fact that waterpipe smoking is a custom and traditional source of entertainment for many families, and women can experience it more easily as a leisure time activity in family gatherings both indoors and outdoors compared to cigarette smoking, which are considered taboo, particularly among older women in Iranian culture (33) and other Middle Eastern countries (1, 5).

Developing effective prevention strategies, particularly among young and high-risk populations, would be a useful approach to controlling waterpipe smoking growth. The authors of a recent review concerning waterpipe tobacco smoking control policies reported that little attention is paid to waterpipe policies overall. Most of the countries with guidelines to control this issue are from the East Mediterranean and the European Regions and around 34.1% were included in high-income economy countries. However, globally, despite the growing appreciation of the importance of waterpipe smoking policies, the number of countries that have regulations in place to address waterpipe smoking comprehensively is not increasing. In particular, some regulations are available. But it was not effective in Iran (34).

Our study had several strengths, including the ability to study daily and occasional smokers in the same population, and the high data quality since data was collected by trained interviewers using a validated questionnaire. We also had detailed information on the main determinants and other confounders, such as different tobacco products. The non-response rate among the participants was not very high (11%), and the main reasons were lack of time or unwillingness to donate a biological sample among referents. Collection of lifetime history of waterpipe smoking is an additional strength (20). There was a wide range of exposure prevalence across Iranian provinces, and people from a wide variety of backgrounds cultures, social groups, and geographical locations were included in the study. Finally, according to previous studies in Western countries, most users are occasional users and try it just for entertainment and not for regular and daily use. Our study also included a good number of this type of user. Thus, our findings on occasional users may be relevant to water pipe smokers from Europe or North America, who are young and smoke water pipe occasionally in public settings.

Also, our study suffers from some limitations. We used a control group of a primary case–control study, and this group was matched according to case group characteristics, particularly age and gender, so the study subjects were not a representative sample of the underlying population, especially for those under 30 years of age. We therefore suggest that future studies should include people of all ages. The number of water-pipe smokers, though relatively large, was small in specific subgroups, such as daily and occasional users. In addition, future studies should investigate detailed aspects of waterpipe smoking, such as the duration of the water pipe session, which were not adequately addressed in our study. Finally, information on waterpipe smoking was self-reported and therefore related to memory and may suffer from recall bias.

In conclusion, waterpipe smoking is associated with younger age, low education and socioeconomic status, alcohol drinking, cigarette and opium users. These findings provide valuable information for stakeholders and policymakers in designing awareness and prevention programs to target specific populations at risk. Furthermore, the results of the study suggest that the findings on occasional waterpipe smoking may be generalized to the younger age group of waterpipe smokers in Western countries. For future studies, longitudinal studies to examine the temporal relationship between risk factors and waterpipe smoking initiation or cessation could be useful and also investigating the effectiveness of interventions and prevention programs targeted at waterpipe smoking is crucial.

## Data availability statement

The raw data supporting the conclusions of this article will be made available by the authors, without undue reservation.

## Ethics statement

The studies involving humans were approved by the Ethics Committee of the National Institute for Medical Research Development (NIMAD) (Code: IR.NIMAD.REC.1394.027). The studies were conducted in accordance with the local legislation and institutional requirements. The participants provided their written informed consent to participate in this study.

## Author contributions

MS: Formal analysis, Methodology, Writing – original draft. AS: Formal analysis, Writing – review & editing. HR: Project administration, Writing – review & editing. MH: Project administration, Writing – review & editing. MM: Project administration, Writing – review & editing. AA-M: Project administration, Writing – review & editing. AN: Project administration, Writing – review & editing. PB: Funding acquisition, Methodology, Supervision, Writing – review & editing. KZ: Funding acquisition, Methodology, Supervision, Writing – review & editing.

## References

[1] Makvandi Z Mostafavi F Bashirian S Zamani-Alavijeh F Kelishadi R. Sociocultural factors contributing to waterpipe tobacco smoking among adolescents and young adult women: a qualitative study in Iran. Int J Qual Stud Health Well-being. (2021) 16:1857043. doi: 10.1080/17482631.2020.1857043, PMID: 33435855 PMC7808745

[2] Momenabadi V Hossein Kaveh PDM Hashemi SY Borhaninejad VR. Factors affecting hookah smoking trend in the society: a review article. Addict Health. (2016) 8:123–35. PMID: 27882210 PMC5115646

[3] Cobb C Ward KD Maziak W Shihadeh AL Eissenberg T. Waterpipe tobacco smoking: an emerging health crisis in the United States. Am J Health Behav. (2010) 34:275–85. doi: 10.5993/AJHB.34.3.3, PMID: 20001185 PMC3215592

[4] Asfar T Ward KD Eissenberg T Maziak W. Comparison of patterns of use, beliefs, and attitudes related to waterpipe between beginning and established smokers. BMC Public Health. (2005) 5:19. doi: 10.1186/1471-2458-5-19, PMID: 15733316 PMC553967

[5] Abdulrashid OA Balbaid O Ibrahim A Shah HBU. Factors contributing to the upsurge of water-pipe tobacco smoking among Saudi females in selected Jeddah cafés and restaurants: a mixed method study. J Family Community Med. (2018) 25:13–9. doi: 10.4103/jfcm.JFCM_3_17, PMID: 29386957 PMC5774038

[6] Adetona O Mok S Rajczyk J Brinkman MC Ferketich AK. The adverse health effects of waterpipe smoking in adolescents and young adults: a narrative review. Tob Induc Dis. (2021) 19:1–31. doi: 10.18332/tid/142521 PMC8534427 34720796

[7] Jawad M Charide R Waziry R Darzi A Ballout RA Akl EA. The prevalence and trends of waterpipe tobacco smoking: a systematic review. PloS One. (2018) 13:e0192191. doi: 10.1371/journal.pone.0192191, PMID: 29425207 PMC5806869

[8] Nemati S Rafei A Freedman ND Fotouhi A Asgary F Zendehdel K. Cigarette and water-pipe use in Iran: geographical distribution and time trends among the adult population; a pooled analysis of national STEPS surveys, 2006-2009. Arch Iran med. (2017) 20:295–301. 28510465

[9] Ukema JB Bagnasco DE Jukema RA. Waterpipe smoking: not necessarily less hazardous than cigarette smoking. Neth Heart J. (2014) 22:91–9. doi: 10.1007/s12471-013-0501-0, PMID: 24307377 PMC3931860

[10] Mohammad-Alizadeh-Charandabi S Mirghafourvand M Tavananezhad N Karkhaneh M. Prevalence of cigarette and water pipe smoking and their predictors among Iranian adolescents. Int J Adolesc Med Health. (2015) 27:291–8. doi: 10.1515/ijamh-2014-0028, PMID: 25470603

[11] Mojahed K Navidian A. The effect of motivational interviewing on craving and dependence on hookah in suburban pregnant women in south east of Iran. Issues Ment Health Nurs. (2018) 39:693–9. doi: 10.1080/01612840.2018.1445325, PMID: 29847190

[12] Roohafza H Sadeghi M Shahnam M Bahonar A Sarafzadegan N. Perceived factors related to cigarette and waterpipe (ghelyan) initiation and maintenance in university students of Iran. Int J Public Health. (2011) 56:175–80. doi: 10.1007/s00038-009-0107-x 20020176

[13] Maziak W Ward KD Eissenberg T. Interventions for waterpipe smoking cessation. Cochrane Database Syst Rev. (2007) 4:CD005549. doi: 10.1002/14651858.CD005549.pub2 17943865

[14] Morovatdar N Poorzand H Bondarsahebi Y Hozhabrossadati SA Montazeri S Sahebkar A. Water pipe tobacco smoking and risk of coronary artery disease: a systematic review and Meta-analysis. Curr Mol Pharmacol. (2021) 14:986–92. doi: 10.2174/1874467213666201223121322 33357208

[15] Mamtani R Cheema S Sheikh J Al Mulla A Lowenfels A Maisonneuve P. Cancer risk in waterpipe smokers: a meta-analysis. Int J Public Health. (2017) 62:73–83. doi: 10.1007/s00038-016-0856-2, PMID: 27421466 PMC5288449

[16] Dadipoor S Kok G Aghamolaei T Heyrani A Ghaffari M Ghanbarnezhad A. Factors associated with hookah smoking among women: a systematic review. Tob Prev Cessat. (2019) 5:26. doi: 10.18332/tpc/110586 32411889 PMC7205165

[17] Cooper M Pacek LR Guy MC Barrington-Trimis JL Simon P Stanton C. Hookah use among US youth: a systematic review of the literature from 2009 to 2017. Nicotine Tob Res. (2019) 21:1590–9. doi: 10.1093/ntr/nty135, PMID: 29961895 PMC6861827

[18] Salles TV de Andrade AG de Oliveira LG. A qualitative study of chronic hookah use in São Paulo. Brazil Subst Use Misuse. (2021) 56:1910–4. doi: 10.1080/10826084.2021.1958857, PMID: 34347562

[19] Moghadam TZ Zandian H Fazlzadeh M Kalan ME Pourfarzi F. Socioeconomic and environmental factors associated with waterpipe tobacco smoking among Iranian adults: a PERSIAN cohort-based cross-sectional study. BMC Public Health. (2023) 23:1295. doi: 10.1186/s12889-023-16176-8, PMID: 37407959 PMC10324125

[20] Hadji M Rashidian H Marzban M Gholipour M Naghibzadeh-Tahami A Mohebbi E. The Iranian study of opium and Cancer (IROPICAN): rationale, design, and initial findings. Arch Iran Med. (2021) 24:167–76. doi: 10.34172/aim.2021.27, PMID: 33878874

[21] Mohebbi E Hadji M Rashidian H Rezaianzadeh A Marzban M Haghdoost AA. Opium use and the risk of head and neck squamous cell carcinoma. Int J Cancer. (2021) 148:1066–76. doi: 10.1002/ijc.33289, PMID: 32895947

[22] Morton J Song Y Fouad H Awa FE Abou El Naga R Zhao L. GATS collaborative group. Cross-country comparison of waterpipe use nationally representative data from 13 low and middle-income countries from the global adult tobacco survey (GATS). Tob Control. (2014) 23:419–27. doi: 10.1136/tobaccocontrol-2012-050841, PMID: 23760609 PMC4145417

[23] Sarrafzadegan N Toghianifar N Roohafza H Siadat Z Mohammadifard N O'Loughlin J. Lifestyle-related determinants of hookah and cigarette smoking in Iranian adults. J Community Health. (2010) 35:36–42. doi: 10.1007/s10900-009-9186-0, PMID: 19866347

[24] Casagrande SS Wang Y Anderson C Gary TL. Have Americans increased their fruit and vegetable intake? The trends between 1988 and 2002. Am J Prev Med. (2007) 32:257–63. doi: 10.1016/j.amepre.2006.12.002, PMID: 17383556

[25] Mohammadkhani S Rezaei JH. Relationship between cigarette and hookah smoking with individual, family and social factors in adolescents. J Sabzevar Univ Med Sci. (2016) 23:262–80.

[26] Jackson D Aveyard P. Waterpipe smoking in students: prevalence, risk factors, symptoms of addiction, and smoke intake. Evidence from one British university. BMC Public Health. (2008) 8:174. doi: 10.1186/1471-2458-8-174, PMID: 18498653 PMC2413225

[27] Abdollahifard G Vakili V Danaei M Askarian M Romito L Palenik CJ. Are the predictors of hookah smoking differ from those of cigarette smoking? Report of a population-based study in shiraz, Iran, 2010. Int J Prev Med. (2013) 4:459–66. PMID: 23671779 PMC3650599

[28] Salloum RG Haider MR Barnett TE Guo Y Getz KR Thrasher JF. Waterpipe tobacco smoking and susceptibility to cigarette smoking among young adults in the United States, 2012-2013. Prev Chronic Dis. (2016) 13:E24. doi: 10.5888/pcd13.150505, PMID: 26890407 PMC4758799

[29] Maziak W Eissenberg T Ward K. Patterns of waterpipe use and dependence: implications for intervention development. Pharmacol Biochem Behav. (2005) 80:173–9. doi: 10.1016/j.pbb.2004.10.026, PMID: 15652393

[30] Chen K-T Chen C-J Fagot-Campagna A Narayan K. Tobacco, betel quid, alcohol, and illicit drug use among 13-to 35-year-olds in I-Lan, rural Taiwan: prevalence and risk factors. Am J Public Health. (2001) 91:1130–4. doi: 10.2105/AJPH.91.7.1130, PMID: 11441745 PMC1446715

[31] Danaei M Jabbarinejad-Kermani A Mohebbi E Momeni M. Waterpipe tobacco smoking prevalence and associated factors in the southeast of Iran. Addict Health. (2017) 9:72–80. PMID: 29299209 PMC5742413

[32] Xuan le TT Van Minh H Giang KB Nga PT Hai PT Minh NT. Prevalence of waterpipe tobacco smoking among population aged 15 years or older, Vietnam, 2010. Prev Chronic Dis. (2013) 10:E57. doi: 10.5888/pcd10.120100, PMID: 23597395 PMC3638612

[33] Kelishadi R Sadry G Zadegan NS Hashemipour M Sabet B Bashardoust N. Smoking, adolescents and health: Isfahan healthy heart program-heart health promotion from childhood. Asia Pac J Public Health. (2004) 16:15–22. doi: 10.1177/101053950401600104, PMID: 18839863

[34] Alaouie H Krishnamurthy Reddiar S Tleis M El Kadi L Afifi A Nakkash R. Waterpipe tobacco smoking (WTS) control policies: global analysis of available legislation and equity considerations. Tob Control. (2022) 31:187–97. doi: 10.1136/tobaccocontrol-2021-056550, PMID: 35241587

